# The Role of Transfer in Designing Games and Simulations for Health: Systematic Review

**DOI:** 10.2196/games.7880

**Published:** 2017-11-24

**Authors:** Derek A Kuipers, Gijs Terlouw, Bard O Wartena, Job TB van 't Veer, Jelle T Prins, Jean Pierre EN Pierie

**Affiliations:** ^1^ NHL Stenden University of Applied Sciences Leeuwarden Netherlands; ^2^ Medical Faculty LEARN University Medical Center Groningen University of Groningen Groningen Netherlands; ^3^ Industrial Design Engineering Delft University of Technology Delft Netherlands; ^4^ MCL Academy Medical Center Leeuwarden Leeuwarden Netherlands; ^5^ Surgery Department Medical Center Leeuwarden Leeuwarden Netherlands; ^6^ Post Graduate School of Medicine University Medical Center Groningen University of Groningen Groningen Netherlands

**Keywords:** transfer, computer simulation, video games, serious games, games for health, fidelity, abstract learning, immersion, metaphor

## Abstract

**Background:**

The usefulness and importance of serious games and simulations in learning and behavior change for health and health-related issues are widely recognized. Studies have addressed games and simulations as interventions, mostly in comparison with their analog counterparts. Numerous complex design choices have to be made with serious games and simulations for health, including choices that directly contribute to the effects of the intervention. One of these decisions is the way an intervention is expected to lead to desirable transfer effects. Most designs adopt a first-class transfer rationale, whereas the second class of transfer types seems a rarity in serious games and simulations for health.

**Objective:**

This study sought to review the literature specifically on the second class of transfer types in the design of serious games and simulations. Focusing on game-like interventions for health and health care, this study aimed to (1) determine whether the second class of transfer is recognized as a road for transfer in game-like interventions, (2) review the application of the second class of transfer type in designing game-like interventions, and (3) assess studies that include second-class transfer types reporting transfer outcomes.

**Methods:**

A total of 6 Web-based databases were systematically searched by titles, abstracts, and keywords using the search strategy (video games OR game OR games OR gaming OR computer simulation*) AND (software design OR design) AND (fidelity OR fidelities OR transfer* OR behaviour OR behavior). The databases searched were identified as relevant to health, education, and social science.

**Results:**

A total of 15 relevant studies were included, covering a range of game-like interventions, all more or less mentioning design parameters aimed at transfer. We found 9 studies where first-class transfer was part of the design of the intervention. In total, 8 studies dealt with transfer concepts and fidelity types in game-like intervention design in general; 3 studies dealt with the concept of second-class transfer types and reported effects, and 2 of those recognized transfer as a design parameter.

**Conclusions:**

In studies on game-like interventions for health and health care, transfer is regarded as a desirable effect but not as a basic principle for design. None of the studies determined the second class of transfer or instances thereof, although in 3 cases a nonliteral transfer type was present.

We also found that studies on game-like interventions for health do not elucidate design choices made and rarely provide design principles for future work. Games and simulations for health abundantly build upon the principles of first-class transfer, but the adoption of second-class transfer types proves scarce. It is likely to be worthwhile to explore the possibilities of second-class transfer types, as they may considerably influence educational objectives in terms of future serious game design for health.

## Introduction

Games and simulations hold the promise of being learning machines [1] because of the ability to build in learning principles. They can harvest unique features to motivate, trigger, and facilitate learning processes, opening up new possibilities for designing learning for health care professionals and patients. With the positive effects on learner motivation and learning outcomes in mind [2-4], educators must think of new ways to make serious subject matter suitable for game play. A transformation of current forms and beliefs on learning may be needed to make a more natural connection between the *serious* and the *game*.

### Transfer

A possible way to make such a connection can be found in thinking in terms of transfer. Although there are a wide variety of viewpoints and theoretical frameworks regarding transfer in the literature, transfer is seldom a starting point in developing serious games. Studies on serious games [5,6] have identified design principles for flow and immersion as major contributors to the gaming experience and presumably beneficial for learning. However, the way games facilitate learning is often regarded as a black box.

From an educational and technological perspective, transfer is a key concept in learning theory and education [7]. The purpose of (medical) education is transfer: the application of skills, knowledge, or attitudes that were or learned in one situation to another context. The concept of transfer is widely recognized, but ample evidence shows that transfer from learning experiences often does not occur. The prospects and conditions of transfer are crucial educational issues.

If we regard games and simulations as learning contexts that can be designed and specifically tailored for (at least a type of) transfer, it seems legitimate to focus attention on how transfer has been taken into account in designing game-like health interventions.

### Two Classes of Transfer

Transfer theory determines two classes of transfer, both encompassing a variety of transfer types [8]. The first class takes the position that the more the learning context resembles the target context, the more likely transfer is to occur. The conditions for transfer are met when the learning experience shares common stimulus properties with the target context. This means that when game or simulation environments try to represent the real world as literal as possible, they aim for first-class transfer. The first class of transfer encompasses instances of literal, specific, nonspecific, vertical, lateral and low-road transfer.

The second class of transfer theories may be harder to grasp. According to Royer [7], figural transfer (belonging to the second transfer class) involves situations where a known complex of ideas, concepts, and knowledge is juxtaposed against some new problem or situation. Figural transfer uses existing world knowledge to think or learn about a particular issue. Clear examples of the usage of figural transfer can be found in figural language such as metaphor or simile. Transfer occurs because of a successful memory search triggered by a figural learning context, assisting in understanding the transfer context. In some situations, the second class of transfer requires a debrief to explicate experiences and connections made. This class encompasses high-road transfer [8].

### Optimizing Transfer Conditions

Games and simulations for health abundantly build on the principles of first-class literal transfer, but the adoption of second-class transfer types has proven to be scarce. In contrast to commercial off-the-shelf games, in serious game design, the usage of mindful abstractions and metaphorical representations is not common practice, despite the fact that it forms a natural fit with the second class of transfer theories. Earlier research has shown [9] that transfer is hard to establish and that the design of education should be key to optimize the conditions under which transfer can occur. Although *transfer of learning* is a well-established concept in the educational domain, the extent to which transfer may guide the development of game-like interventions in health has rarely been explored. This may be especially true for second-class transfer types: optimizing a game-like intervention design to accommodate the principles of figural transfer.

### Fidelity Types

The most visible examples of the designers’ uptake of transfer in game-like interventions are apparent in the application of fidelity types: the way fidelity is used in a game-like intervention or simulation demonstrates the expected road to transfer. A dominant perspective on fidelity in serious game design is that high fidelity is conditional for learning and transfer, corresponding with the first class of transfer.

According to Alexander [10], fidelity has dimensions beyond the visual design—physical, functional, and psychological fidelity [10]. A game or simulation therefore can be low in physical and functional fidelity but can be high in psychological fidelity. It is also possible that a simulation by design is high in functional and physical fidelity but lacks psychological fidelity. In the literature, the degree of fidelity often refers to physical fidelity alone. Therefore, in this study, cases of cognizant design decisions toward lower fidelity types may prove interesting, as they might include second class of transfer types.

### Aim

Focusing on the design of game-like interventions for health and health care, this study aimed to (1) find out whether the second class of transfer is recognized or present as a road for transfer in game-like interventions, (2) review the application of the second class of transfer type in designing game-like interventions, and (3) assess studies that include second-class transfer types reporting transfer outcomes.

## Methods

### Databases and Search Strategy

In total, 6 databases were searched for potentially relevant abstracts: PubMed, Scopus, ERIC, PsycINFO, Information Science & Technology Abstracts, and EMBASE. These databases covered a wide range of published research from the field of health and social care. A combination of search terms were used to identify relevant papers under the following categories: (video games OR game OR games OR gaming OR computer simulation*) AND (software design OR design) AND (fidelity OR fidelities OR transfer* OR behaviour OR behavior), where * represents a wildcard to allow for alternative suffixes. Search strategies were customized for each database. Searches included papers published between database inception and October 2016. The search was conducted between October 3, 2016 and October 21, 2016.

### Study Selection and Inclusion and Exclusion Criteria

We included studies that discussed either digital simulations or games designed for health providers or on health topics. We included only original reports or papers that (1) addressed the design of a serious game or digital simulation; (2) involved an empirical study, either piloting a game-like intervention or validating the aspired effects; or (3) otherwise focused on a newly developed game or simulation, created specifically for the study in question. Papers were included when title and abstract were considered to be at least indicative of the presence of second class of transfer. Papers meeting any of the above criteria were selected for full-text screening.

The following exclusion criteria were used for full-text screening: (1) non–peer-reviewed papers such as abstracts, conference posters, or trade journals; (2) full text not available; (3) language other than English and Dutch; (4) papers that referred to transfer as transfer of data or disease; (5) not sufficient information; (6) repurposed commercial off-the-shelf games; (7) low fidelity as a means to reduce production costs; (8) nondigital games and simulations; and (9) papers using high fidelity solely as a description of the artifact rather than as a founded design decision. Also, in our screening, we considered the transfer class in relation to the fidelity type: high fidelity as a means for achieving literal transfer led to exclusion.

### Screening Process

After removing the duplicates, the papers were screened based on title and abstract using Rayyan [11]. In total, 2 reviewers (DK and GT) independently reviewed the title and abstract for relevance against the formulated inclusion/exclusion criteria. Papers were only included on the agreement of both DK and GT; a third reviewer (BW) resolved any disagreements. The degree of agreement was calculated by a kappa statistic. Full-text papers were retrieved after this step. Both reviewers (DK and GT) reviewed each included full-text article. Disagreements in this stage about inclusion were discussed until an agreement was reached. Finally, to check whether any eligible paper had been overlooked during the review process, our check included studies’ references for additional papers.

## Results

### Search Results

**Figure 1:**
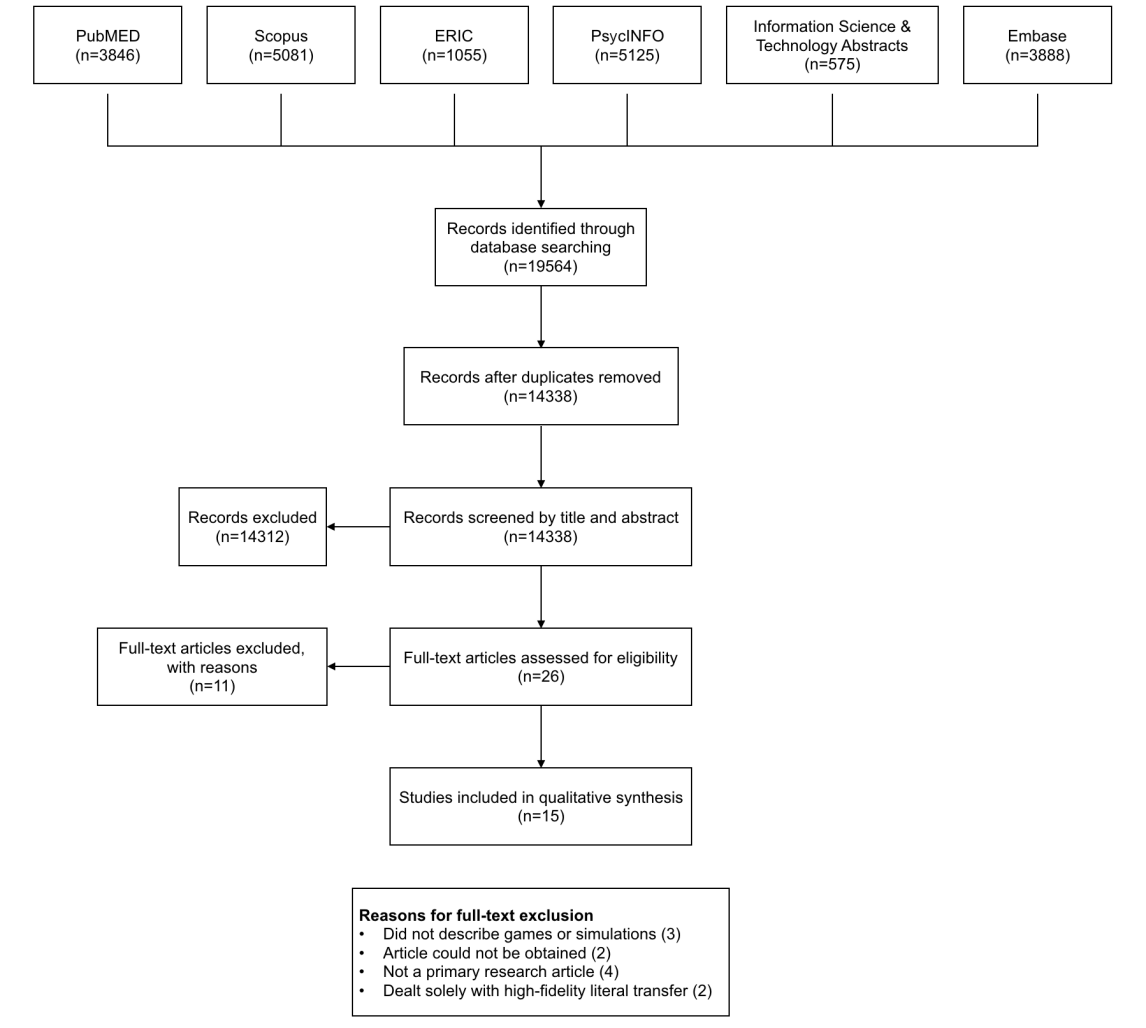
Flowchart of the results of the initial searches, screening, and selection processes.

Our initial search yielded 19,564 records. After removing all duplicates (5226), 14,338 records remained for title and abstract screening, leaving 26 potential suitable papers for full-text assessment. We used Cohen kappa to assess the interrater reliability of paper inclusion. We found good agreement between the 2 reviewers (ϰ=.78, 95% CI 0.655-0.883). A total of 11 papers were excluded at full-text screening for various reasons. The total number of included papers is therefore 15. See Figure 1 for a flowchart of the results of the initial searches, screening, and selection processes. Table 1 shows an overview of included studies.

### Second-Class Transfer in Game-like Interventions for Health and Health Care

We studied the full-text papers on how transfer was regarded and described in serious games or simulations. All 15 studies mentioned transfer in the initial concept of the design and described forthcoming consequences, mostly expressed in terms of fidelity. Although we assumed that the second class of transfer would be identified in varying ways, we found several other reasons to use abstract concepts and low fidelity. In the following section, we have categorized the papers, based on similarities in conjoining characteristics.

#### Reducing Cognitive Load

Out of the selected studies, 3 [12,17,24] questioned the necessity of high fidelity to achieve transfer. The basic theory is that high-immersive gaming environments decrease learning outcomes. The studies argue that reducing complexity prevents extraneous cognitive load. In these situations, low fidelity and deliberate abstractions are aiming—by design—for managing the trainees’ working memory capacity. This is grounded in the cognitive load theory [27].

#### Motor and Spatial Skills Training in Metaphorical Contexts

In our initial selection process, papers that presented literal transfer axiomatically were excluded. Out of the included studies, 3 [13-15] were regarded more closely because they use metaphorical game environments, possibly indicating the presence of second-class transfer types. These game-like interventions were designed for skills training (ie, laparoscopic surgery, spatial cognition skills, lifting and transfer techniques). In these scenarios, emphasis is placed on a high degree of validated functional fidelity, aimed at faithfully mimicking the desired skills. These games facilitate first-class low-road transfer by automating motor and spatial skills, hosted by low physical fidelity metaphors.

For example, the game *Underground* carefully mimics basic laparoscopic skills, including custom-made laparoscopic tool shells. The movements in the game are carefully calibrated to faithfully represent actual laparoscopic skills. These skills are acquired in a literal way. It is noteworthy that the tools and movements share high functional fidelity properties, whereas the physical fidelity is low or even nonexistent. The same goes for *iLift*, where the lifting and transfer techniques maintain a mimetic correspondence to real-world tasks, although the game metaphor encompasses physical and psychological fidelity: catching sheep or helping little robots escape from a mine shows no medical content. These games use metaphorical contexts to host meaningful play for training skills.

#### Situational Games

Pervasive game design provides a different approach toward transfer. Of the papers, 2 [19,25] advocate pervasive games, where fusing the virtual world with the real-world positions them in between the first and second class of transfer. One could argue that situational games seek to provide what we like to call *blended transfer* by emphasizing context awareness in a true-to-life experience on the one hand and on the other hand adding virtual game elements. Both studies conclude by accentuating the promise of pervasive game play for transfer of knowledge [25] and transfer of behavior [19] but provide no implications for the design of virtual elements for future pervasive games.

**Table 1:** Details of included papers.

Author	Title	Transfer class^a^	Fidelity and transfer rationale	Year
Dankbaar, Alsma, Jansen, Van Merrienboer, Van Saase, and Schuit [12]	An experimental study on the effects of a simulation game on students’ clinical cognitive skills and motivation	First	Low fidelity, reducing cognitive load	2016
Kuipers, Wartena, Dijkstra, Terlouw, van T Veer, Van Dijk, Prins, and Pierie [13]	iLift: A health behavior change support system for lifting and transfer techniques to prevent lower-back injuries in healthcare	First	Low-road transfer, skill automatization, metaphorical	2016
Jalink, Gores, Heineman, Pierie, and Ten Cate Hoedemaker [14]	Face validity of a Wii U video game for training basic laparoscopic skills	First	Low-road transfer, skill automatization, metaphorical	2015
Connors, Chrastil, Sanchez, and Merabet [15]	Action video game play and transfer of navigation and spatial cognition skills in adolescents who are blind	First	Low-road transfer, spatial recognition	2014
Rosenberg, Baughman, and Bailenson [16]	Virtual superheroes: using superpowers in virtual reality to encourage prosocial behavior	Second	Figural, metaphorical	2013
Schrader and Bastiaens [17]	The influence of virtual presence: effects on experienced cognitive load and learning outcomes in educational computer games	First	Low fidelity, reducing cognitive load	2012
De Freitas and Dunwell [18]	Understanding the representational dimension of learning: the implications of interactivity, immersion and fidelity on the development of serious games	Second	Figural, metaphorical	2012
Knoll and Moar [19]	The space of digital health games	Blended	Locative, situational	2012
Rooney [20]	A theoretical framework for serious game design: exploring pedagogy, play, and fidelity and their implications for the design process	Blended, both	Abstraction, situational	2012
Toups, Kerne, and Hamilton [21]	The team coordination game: zero-fidelity simulation abstracted from fire emergency response practice	Second	Nonmimetic, abstraction	2011
Hochmitz and Yuviler-Gavish [22]	Physical fidelity versus cognitive fidelity training in procedural skills acquisition	First	Cognitive fidelity, skill acquisition	2011
Stone [23]	The (human) science of medical virtual learning environments	First	Cost reduction	2011
Wood, Beckmann, and Birney [24]	Simulations, learning, and real world capabilities	First	Low fidelity, execution skills, reducing cognitive load	2009
Markovic, Petrovic, Kittl, and Edegger [25]	Pervasive learning games: a comparative study	First	Situational	2007
Alessi [26]	Fidelity in the design of instructional simulations	Both	Varying fidelity under conditions	1988

^a^Refers to the aspired transfer type described or sought after with the game-like intervention.

### The Application of the Second Class of Transfer

Of the studies, 3 describe game designs applying the second class of transfer, and one study [16] describes a video game to stimulate prosocial behavior. It examines how playing an avatar with superhero abilities increases prosocial behavior in the real world *.* The study indicates that the in-game superhero metaphor leads to greater helping behavior outside the game. The game therefore builds on the second class of transfer, although the study does not explicate design considerations regarding transfer.

De Freitas et al [18] describe *Re-Mission*, a video game designed for young people with cancer to encourage them to take their medication. The game metaphor, where the player has to combat cancer cells, seeks to reinforce behavioral change toward medication use. *Re-Mission* fits the figural transfer class as the in-game representation of the illness and the power to conquer this illness are metaphorical rather than literal [18]. The game play shows little physical or functional fidelity to real-world processes, and measured effects can only be explained in terms of changed mental conceptions, referring to an instance of second-class (figural) transfer. However, the design considerations were not elucidated either in this study.

The third study [21] describes the *Team Coordination Game*, a simulation to practice team coordination during fire emergency response situations. The *Team Coordination Game* is a simulation that offers a game environment that requires the use of effective team communication skills, without concrete elements of the mimicked environment. This nonmimetic game offers a two-dimensional environment that shows low-fidelity to real-life fire emergency environments. In the game, 3 avatars in the role of *seeker* are searching for specific goals, while avoiding threats. A player in the role of coordinator directs the seekers based on observing the environment from a different angle. Limited game time creates a certain amount of stress and pushes the players to work effectively. The study suggests that players were able to restore learned behaviors in communication and stress management in an alternative environment, remixing and repurposing them, suggesting a transfer effect.

Although the game offers a so-called zero-fidelity physical environment, it uses communication instruments that have the same characteristics as real-world radios. This implies at least a modicum of functional fidelity. Furthermore, the game is based on communication strategies and stress levels from real-world fire emergency situations, which suggests some level of psychological fidelity. The *Team Coordination Game* simulation study offers clear design implications, labeling and elaborating on abstraction from reality as a guiding principle, which differs from the other studies included.

### Psychological Fidelity

In total, 7 studies [12,18,20-23,26] mention *psychological fidelity* as, if not *the* most, an important design parameter in serious games and simulations. In addition, these studies claim that representing the real world as literal as possible is less important for learning. The definition of psychological fidelity in these studies varies slightly [22], but all studies mention the abstraction of certain real-world concepts and a process of recontextualization. Of the studies considered, one [21] added *suspension of disbelief* as an important characteristic of psychological fidelity: one’s temporary allowance to believe something that is not true. Despite the fact that the second class of transfer is not explicitly stated in those studies, they implicitly confirm the second class of transfer as a promising concept in serious game design for learning.

### Effects of Design for Figural Transfer

The virtual superhero study [16] only reported a transfer effect just after playing the game. The study did not cover long-term effects but showed in an experimental 2×2 design that participants (n=60) in the flying superhero condition displayed significantly increased prosocial behavior compared with participants who were in the helicopter condition. The study mentions several possibilities for the differences found between the testing conditions: different experiences of immersiveness, involvement versus observation discrepancy, and primed concepts and stereotypes related to superheroes in general.

The study reporting on *Re-Mission* [18] did not elaborate on the efficacy of the intervention. Another study [28] focused in greater detail on the transfer effects of *Re-Mission* and found that playing the game increased young cancer patients’ feelings of self-efficacy or beliefs in their own ability to control and cope with the disease. A randomized trial with 197 intervention group participants showed a significant increase in cancer-related knowledge and self-efficacy scores and offers empirical support for the efficacy of a game-like intervention in improving behavioral outcomes in adolescents and young adults with cancer.

Using a mixed-method approach, the *Team Coordination Game* [21] also reported some transfer effect, in addition to an in-game effect (n=64). The study suggested that players were not only able to restore learned behaviors in communication and stress management in an alternative environment but also capable of remixing and repurposing them. The article—in several substudies—describes a variety of positive effects on communication and organizational skills, carried over from the game environment to live training.

## Discussion

To our knowledge, this is the first review to explore the aspired transfer in designing game-like interventions in health. We tried to find and describe examples of the application of second-class transfer types by answering 3 research questions, discussed below.

### Design for Transfer in Health

We tried to determine whether the second class of transfer types is recognized or present as a road for transfer in game-like interventions for health. In our initial search, we expected to find studies in which thinking about a desired transfer outcome would form a guiding principle in the design of game-like interventions. Moreover, clearer distinctions in suitable transfer types and established examples of figural transfer (or forms thereof) were anticipated. Both assumptions were proven wrong, and we had to broaden our inclusion criteria to capture studies regarding design considerations, including transfer.

Our results show that transfer is mainly mentioned as a desired outcome, not as a guide in the design process. The appearance of most included game-like artifacts can be explained by the designer’s fidelity approach. As obvious as this seems, this fidelity approach also expresses assumptions about the way the transfer is expected to take place. As described before, we found several reasons for choosing low fidelity over high fidelity and vice versa. As none of the studies were designed for achieving transfer via a specific type or class of transfer, the question arises why the *design for transfer* perspective has received no attention.

By nature, design-centered research focuses more on the design itself and puts less emphasis on the eventual aspired outcome. Although it is too strong to state that the design itself of game-like interventions in health is not taken into account in thinking about desirable transfer outcomes, our search results show that describing the game-like interventions in terms of transfer variables is uncommon. One might argue that the design of a drug is essential to its workings and that the same principle applies for game-like interventions. The design of the artifacts as exercised in the virtual superhero game [16], *Re-Mission* [18], and the *Team Coordination Game* simulation [21] is intentional and differs strongly from game-like interventions as *Underground* [14] or *Digital Economy* [25]. These differences arise from a broad and ill-defined range of variables but inevitably reveal the designer’s intent with regard to how the intervention should carry over the effect. Herein lies the rationale for design for transfer.

### The Presence of the Second Class of Transfer

As described, we searched for particular examples of aspired transfer in the second class of transfer types, and found none. In 3 studies, the reported effects can only be explained via the road of a second-class transfer type but are described in other terminology. Most studies report about psychological fidelity [12,18,20-23,26], virtual presence [17], and immersion [16,18] as important conditions for desired outcome.

An interesting observation is that the included papers show that functional and physical fidelity can be high or low for varying, well-founded reasons and that psychological fidelity is regarded as a variable that preferably should be high. The *Team Coordination Game* simulation [21] adopts a different position in stating that the gaming artifact has zero psychological fidelity. However, the way deliberate abstractions are described and how these resulted in the design of the game itself strongly suggests second-class transfer.

The 3 studies we identified exemplifying an instance of figural transfer introduced a metaphorical approach with recontextualized fidelity types. These game metaphors seem to address and replace both high functional and physical needs as well as promote immersion. At this point, we hypothesize that figural transfer builds upon immersion or virtual presence and subsequent suspension of disbelief [21]. In more abstract game-like interventions, metaphors provide a storyline, a context, and a reason for engaging in play. In this way, psychological fidelity is reappointed by the concept of suspension of disbelief, instigated by the metaphor itself.

### Second-Class Transfer Outcomes

As the literature on transfer has consistently confirmed, long-term transfer effects are hard to measure. This might be particularly the case for the second class of transfer. All 3 examples report transfer effects, albeit short term and only vaguely proven. As second-class transfer is the result of the effects interventions trigger in one’s head, the transfer outcomes are individual, often nonlinear, and even unpredictable if the second class is not implemented with due care. Precisely because of this, we anticipated more conscious and elucidated design examples.

### Limitations

Although this review is based on an extensive search of a large number of health and computer science databases, we hardly found any studies of second-class transfer types in game-like interventions for health. Studies tend to focus on the effectiveness of game-like interventions and the research methods used, not on design factors that lead or contribute to measured effects. Due to the very few direct hits, we focused on the subconscious application of the second class of transfer types by thoroughly screening titles and abstracts. The papers that were included were subject to interpretation, discussion, and consensus of the reviewers (DK, GT, and BW). To counteract subjectivity, papers were independently reviewed by 2 reviewers (DK and GT) and were only included on consensus from both reviewers. Remaining conflicts between the reviewers were resolved by the third reviewer (BW).

### Conclusions

Studies about serious games and game-like interventions for health do not provide a conscious rationale for designing the artifacts for optimizing transfer conditions. We did not find any example of a game-like intervention that was the result of a cognizant design process focusing on transfer outcomes. In general, we found that definitions of low and high fidelity form the strongest influencers on the design of artifacts, mostly exemplified in visual quality or a true-to-life approach. High fidelity was aspired to for its first class, literal transfer aspects without exception. None of the studies explained second class of transfer or instances thereof, although in 3 instances, implicit design choices suggested otherwise. It is notable that studies on game-like interventions for health do not elucidate the design choices made, as they bridge the designer’s intent and the aspired transfer outcome.
